# Electroacupuncture Inhibition of Hyperalgesia in Rats with Adjuvant Arthritis: Involvement of Cannabinoid Receptor 1 and Dopamine Receptor Subtypes in Striatum

**DOI:** 10.1155/2013/393460

**Published:** 2013-05-25

**Authors:** Yin Shou, Yang Yang, Ming-Shu Xu, Ying-Qian Zhao, Lin-Bao Ge, Bi-Meng Zhang

**Affiliations:** ^1^Shanghai First People's Hospital, School of Medicine, Shanghai Jiao Tong University, Shanghai 200080, China; ^2^Neurobiology Laboratory of Brain Shanghai Research Institute of Acupuncture and Meridians, Shanghai 200030, China; ^3^Shaanxi College of Traditional Chinese Medicine, Xianyang 712046, China; ^4^Shanghai Research Institute of Qigong, Shanghai 200030, China; ^5^Shanghai Research Center of Acupuncture and Meridians, Shanghai 201203, China

## Abstract

Electroacupuncture (EA) has been regarded as an alternative treatment for inflammatory pain for several decades. However, the molecular mechanisms underlying the antinociceptive effect of EA have not been thoroughly clarified. Previous studies have shown that cannabinoid CB1 receptors are related to pain relief. Accumulating evidence has shown that the CB1 and dopamine systems sometimes interact and may operate synergistically in rat striatum. To our knowledge, dopamine D1/D2 receptors are involved in EA analgesia. In this study, we found that repeated EA at Zusanli (ST36) and Kunlun (BL60) acupoints resulted in marked improvements in thermal hyperalgesia. Both western blot assays and FQ-PCR analysis results showed that the levels of CB1 expression in the repeated-EA group were much higher than those in any other group (*P* = 0.001). The CB1-selective antagonist AM251 inhibited the effects of repeated EA by attenuating the increases in CB1 expression. The two kinds of dopamine receptors imparted different actions on the EA-induced CB1 upregulation in AA rat model. These results suggested that the strong activation of the CB1 receptor after repeated EA resulted in the concomitant phenomenon of the upregulation of D1 and D2 levels of gene expression.

## 1. Introduction

More than 40 disorders have been endorsed by the World Health Organization (WHO) as conditions that can benefit from acupuncture treatment. Acupuncture has been used for several years in the treatment of acute and chronic pain. Thus, of the 3975 acupuncture research articles that have been published from 1991 to 2009, 1647 (41%) focus on pain and analgesia [1]. According to the latest reports in the American journal of Nature Neuroscience, acupuncture has been found to cause the human body to release some natural painkillers [2]. In addition, arthritis-induced hyperalgesia and allodynia have been shown as suppressed by repeated EA in some animal experiments [3]. Several processes have been proposed to explain the EA effects, primarily the pain relief effects. One of the main processes of the pain relief effects is to stimulate the central nervous system to release neurotransmitters or neuromodulators, such as opioid peptides, serotonin, and noradrenaline, into muscles, spinal cord, and brain. These substances can either change the degree of pain scale or promote the release of some other chemicals, such as neurohormones, that influence the neuroimmune system [4]. Taken together, acupuncture analgesia involves a comprehensive course of multichannels, multilevels, and multilinks. Acupuncture signals and the pain transduction channels of the central nervous system have obvious overlapping parts, and thus, acupuncture may influence pain signal transduction in the central nervous system [5]. In recent years, more and more studies have been conducted to discover the neurobiological mechanisms of acupuncture analgesia. 

 The endocannabinoid system is of great physiological significance in many aspects. In particular, endocannabinoids play an important role in pain modulation [6]. Earlier studies have demonstrated that the cannabinoid receptor system has therapeutic potential in curing inflammatory diseases, such as rheumatoid arthritis [7]. Electroacupuncture (EA) has been suggested by some researchers to be able to modulate neural responses by decreasing the release of gamma-aminobutyric acid in the brain, most likely through a presynaptic cannabinoid receptor 1 (CB1) mechanism [8]. Given the analgesic effects of EA and the importance of the endocannabinoid system in pain modulation, we hypothesized that the action of EA on inflammatory pain may be attributable to increases in the expression of the CB1 receptor. In order to verify this hypothesis, we established a complete Freund's adjuvant arthritis (AA) rat model and observed the effects of EA at the Zusanli (ST36) and Kunlun (BL60) acupoints on the arthritis and the expression of CB1. In addition, the paw withdrawing latency (PWL) of the AA rats in response to noxious heat was analyzed as an indicator of the thermal pain threshold (PT). We chose to observe the above acupoints because of their common use in acupuncture analgesia [9, 10]. Previous studies have shown that cannabinoid CB1 receptors of the rat corpus striatum, including the nucleus accumbens and caudate nucleus, interact with the dopamine system [11–13] and that dopamine D1/D2 receptors are involved in EA analgesia [14]. Therefore, we also monitored the cross-modulation between CB1 and dopamine D1 and D2 receptors in the analgesic effects of repeated EA. 

## 2. Materials and Methods

### 2.1. Animals

60 adult male Sprague-Dawley (SD) rats (age, 5 weeks old; weight, 180 ± 20 g; Shanghai Laboratory Animal Center, Chinese Academy of Sciences, Shanghai, China) were raised in groups of 4–6 per cage under controlled conditions (23 ± 1°C; relative humidity, 50% ± 10%, 12-h/12-h alternating light/dark cycles; and food and water ad libitum). All animals were handled with care to prevent infection and minimize stress. They all exhibited normal PWL values (8–11 s).

### 2.2. Environmental Adaptation and Grouping

**Figure 1:**
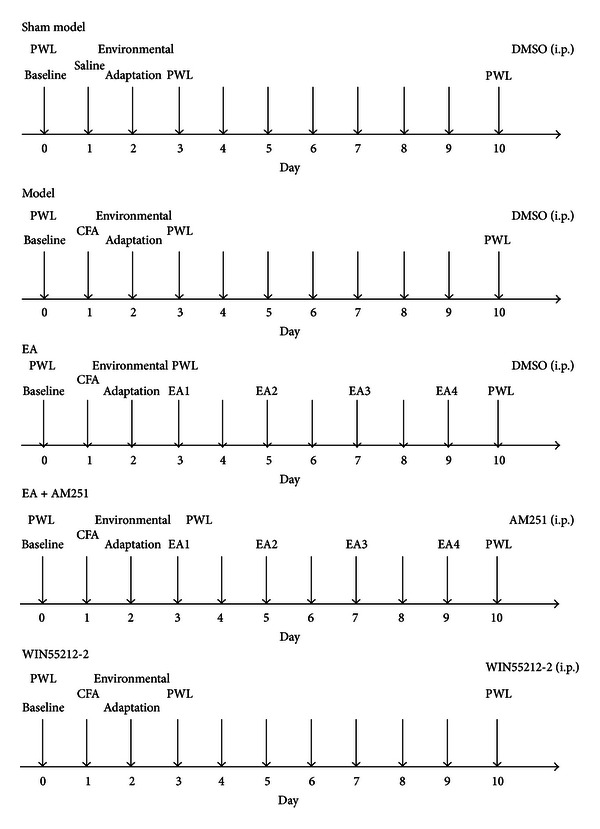
Experimental protocol: 60 rats were used with 12 rats each in 5 groups.

60 SD male rats were randomized into the following groups with 12 rats per group: sham model (injection of saline), model (injection of Complete Freund's Adjuvant (CFA) to induce arthritis), EA (injection of CFA followed by EA stimulation at ST36 and BL60), EA + AM251 (injection of CFA followed by EA stimulation at ST36 and BL60 and an injection of the CB1-selective antagonist AM251), and WIN55212-2 (injection of CFA followed by an injection of the potent cannabinoid receptor agonist WIN55212-2). At least one week before the experiment, all rats were habituated to a plastic chamber. All of the rats, except for the sham-model group, underwent AA model establishment on the 1st day, and they were taken for environmental adaptation on the 2nd day. On the 3rd day, the rats were subjected to PT measurements. After that, EA (2/100 Hz, 30 s, 1.0, −2.0, −3.0 mA) was applied to the “Zusanli” (ST 36) and “Kunlun” (BL 60) acupoints for 20 min, once every other day starting from the 4th day, for 4 sessions. The other groups of rats were maintained within the same small cages with five holes for their four limbs and their tail. On the 10th day, the rats in the relevant groups were intraperitoneally injected with AM251 (1 mg/kg) [15] or WIN55212-2 (2 mg/kg) [16]. The rest of the rats were intraperitoneally injected with an equal volume of 10% dimethyl sulfoxide according to their weight. One and a half hour later, all rats were decapitated, and specimen preparations were made after the last PT measurements. All animal experiments and protocols were approved by the Committee on the Ethics of Animal experiments of Shanghai University of Traditional Chinese Medicine (approval ID: 08001) and were conducted in accordance with the National Institutes of Health Guide for the Care and Use of Laboratory Animals and the International Association for the Study of Pain's (IASP) guidelines for pain research [17]. We conducted a randomized, controlled experiment with blinded evaluation and statistical analyses of the results. A flow chart of the study protocol is shown in the schematic diagram in Figure 1. 

### 2.3. Reagents

Complete Freund's adjuvant (CFA) was used to induce inflammatory pain. The CB1-selective antagonist AM251 and the potent cannabinoid receptor agonist WIN55212-2 were used to interfere with the natural processes of the cannabinoid CB1 receptors. All of the above reagents were purchased from Sigma-Aldrich Co. LLC (St. Louis, MO, USA).

### 2.4. Instruments

 An IITC Model 336GT paw/tail stimulator analgesia meter was purchased from IITC Life Science Inc. (Woodland Hills, CA, USA). Han's acupoint nerve stimulator LH202H was bought from Beijing Huawei Electronic Industry Development Co., Ltd (Beijing, China). The rodent brain matrix was purchased from Analytical Scientific Instruments (Richmond, CA, USA). 

### 2.5. Induction of the AA Rat Model by CFA

The AA rat model was established on the 1st day of the experiment. It was induced as described previously [18]. Rats were anesthetized with 10% chloral hydrate (40 mg/kg, i. p.).The skin around the site of the injection was sterilized with 75% alcohol. A 25-gauge needle was inserted vertically to penetrate the skin and then turned distally to insert it into the left ankle articular cavity of the hind paw from the gap between the tendo calcaneus and the lateral malleolar fossa until an obvious decrease in resistance was felt. Then, a dose of 0.05 mL of CFA (1 mg/mL), which contained 0.05 mg of heat-killed and dried *Mycobacterium tuberculosis* in 85% paraffin oil and 15% mannide monooleate, was administered to this site. Rats in the sham group were injected with an equal volume of sterile normal saline instead of CFA. CFA is a strong and effective inflammation-inducing agent. It can induce obvious local inflammatory pain that lasts for a long period. A Complete Freund's adjuvant arthritis (AA) model can better reflect the essence of pain and the analgesic courses. It is thought that the exaggerated pain results from peripheral sensitization due to an increase in the sensitivity of the nociceptive primary afferent neurons and central sensitization due to the hyperexcitability of nociceptive neurons in the central nervous system [19–22].

### 2.6. Blinded PWL Method

Thermal hyperalgesia was evaluated by measuring the latency of the PWL according to a previously described method [23]. The thermal stimulus was produced with the IITC model 336GT paw/tail stimulator analgesia meter. PWL was taken as an indicator of PT. An automatic 20 s cutoff was utilized to avoid tissue injury. Unrestrained and conscious rats were placed in the clear plastic chambers (12 × 12 × 18 cm) on an elevated surface, and they were allowed to habituate to the testing apparatus for 20 min. Then, the radiant heat that was produced by a strong light beam that was applied to the left hind paw near the toes. The time from onset of the radiant heat application to paw withdrawal was regarded as PWL. Each animal was tested three times with an interval of 15 min, and the average was chosen as the baseline of PT. Those animals for which the baselines were higher than 11 s or lower than 8 s were excluded from the experiment. In addition, to control for potential bias, the investigators who performed the behavioral tests were blinded to the actual EA procedure.

### 2.7. EA Treatment

**Figure 2:**
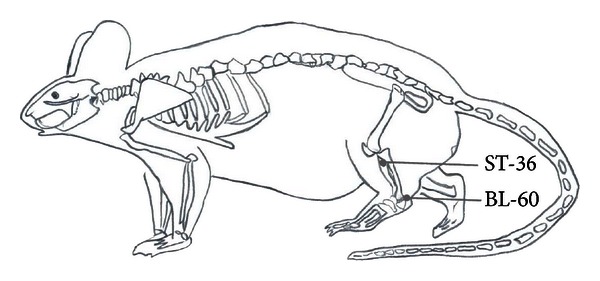
Schematic diagram indicating the selected acupoints, Kunlun (BL 60) and Zusanli (ST36), which correspond to the equivalent acupoints in humans.

On the 3rd day, EA stimulation was performed. Sterilized disposable stainless-steel acupuncture needles (0.3 mm in diameter and 25 mm in length; Suzhou Medical Appliance Factory, Suzhou, China) were inserted to a depth of 7 mm into the left hind limb at ST36 and to a 2 mm insertion depth at BL60. As illustrated in Figure 2, Zusanli (ST36) is located 5 mm lateral and distal to the anterior tubercle of the tibia, and Kunlun (BL60) is located at the ankle joint level between the external malleolus and the tendo calcaneus in the hind limb. The needles that were inserted into the acupoints were connected to an EA stimulator. The stimuli were generated with a constant current programmed pulse generator and applied for 30 min. The electric stimuli were set to be square waves with a, 0.5 ms width, and a frequency of 2 or 100 Hz. The intensity was adjusted so that local muscle contractions were seen. Intensities of 1.0, 2.0, and 3.0 mA were applied for 10 min each. EA treatment was given every other day for 7 sessions in total. In order to examine the possible effects of EA, the other groups of rats were placed in the same apparatus, and they had needle insertions into the same acupoints. However, no electrical current was applied to these groups of rats [24].

### 2.8. Specimen Preparations

All rats were anesthetized and decapitated immediately after the PT measurements. The brains were removed from the skull and then transferred, ventral side up, to an ice-cold Rodent Brain Matrix with 0.5 mm spacing (this process required 1–1.5 min). The nucleus accumbens and striatum were taken from the adjacent slice that was approximately 2.0 mm to 1.5 mm anterior to the bregma with two thin, double-edged razor blades. Following the removal from the matrix, the 2.0 mm brain slices were placed flat onto an ice-cold dissection stage, and specific regions were dissected according to anatomical landmarks as described below. The brain tissues that were isolated from the right and left sides were rapidly frozen in liquid nitrogen and stored at −80°C [25–27].

### 2.9. Fluorescent Quantitative Reverse Transcription-Polymerase Chain Reaction (FQ-PCR) Analysis

Total RNA was prepared from treated frozen brain tissues that were treated with TRIzol extraction reagent (Shanghai UniBi Tec, Shanghai, China) and reverse transcribed with an M-MLV first-strand cDNA synthesis kit (Promega Corporation, Madison, WI, USA) and oligo(dT) primer, as recommended by the manufacturer. A 50 *μ*L PCR reaction contained 10 *μ*L of 5X Taq PCR Master Mix buffer, 0.5 *μ*L of upstream primer, 0.5 *μ*L of downstream primers, 0.5 *μ*L of TaqMan fluorescence probe, 1 *μ*L of taq polymerase, 0.5 *μ*L of dNTPs, 32 *μ*L ddH_2_O, and 5 *μ*L of first-strand cDNA. Quantitative PCR was performed with an iCycler iQ real-time PCR system (Bio-Rad Laboratories, Inc., Hercules, CA, USA). Amplified products were detected with SYBR Green PCR Master Mix (Toyobo Co., Ltd., Osaka, Japan). The sequences of the primer pairs that were specific for each gene are shown in Table 1. The PCR was initially denatured at 50°C for 2 min, which was followed by 40 PCR cycles of 95°C for 5 min, 95°C for 15 s, and 60°C for 45 s. The fold changes in expression were calculated relative to the expression of glyceraldehyde 3-phosphate dehydrogenase (GAPDH), which was used as an endogenous control for normalization. These mRNA levels were quantified by FQ-PCR based on TaqMan technology with the ABI PRISM 7500 Sequence Detection System (Life Technologies Corporation, Grand Island, NY, USA). Fluorescence was measured for each amplification cycle, and the data were analyzed with the 2^−ΔΔCT^ method for the relative quantification of expression. Reactions were performed in duplicate [28].

### 2.10. Western Blot Analysis

The brain tissues were collected and stored for FQ-PCR. Each sample was weighed and homogenized in 100 mL of RIPA Lysis Buffer (Beyotime Institute of Biotechnology, Nanjing, China) containing 0.01 M Tris-HCl buffer (pH 7.6), 0.25 M sucrose, 0.1 M NaCl, 1 mM ethylenediaminetetraacetic acid, and 1 mM phenylmethyl sulfonyl fluoride at 4°C. The supernatant was centrifuged at after 12,000 rpm for 10 min and then examined with western blotting. The pellet was discarded, and the protein concentrations of the supernatant were determined with an Enhanced BCA Protein Assay Kit (Beyotime Institute of Biotechnology). In order to quantify the CB1 protein levels, 40 *μ*g of total protein of each tissue was processed with 2x Tricine-SDS-PAGE loading buffer (Beyotime Institute of Biotechnology), separated on 10% gels with sodium dodecyl sulfate-polyacrylamide gel electrophoresis and then electrotransferred at 300 mA to Immun-Blot polyvinylidene fluoride membranes (EMD Millipore, Corporation, Billerica, MA, USA) for 1 h. Membranes were blocked in Tris-buffered saline with phosphate-buffered saline containing 5% nonfat dried milk and 0.1% Tween 20 for two hours at room temperature. The blocked membranes were incubated overnight at 4°C with the rabbit anti-cannabinoid CB1 receptor polyclonal antibody (1 : 250, Sigma-Aldrich Co, LLC), or the rabbit anti-GAPDH-horseradish peroxidase polyclonal antibody (1 : 4,000, Jingmei, Shanghai, China), which were diluted in Tris-Buffered Saline with Tween (TBST) containing 5% bovine serum albumin. Blots were washed extensively in TBST and incubated with goat anti-rabbit IgG that was conjugated to horseradish peroxidase (1 : 10,000, Immunology Consultants Laboratory, Inc., Portland, OR, USA) in TBST/1.25% bovine serum albumin for 1 h at room temperature. Immunocomplexes were visualized with enhanced chemiluminescence detection reagents (Beyotime Institute of Biotechnology) on X-ray films according to the manufacturer's protocol. The GAPDH band was used as an internal control. The relative intensities of each band on the western blots were measured with a computer-assisted imaging analysis system (Quantity One Software, Bio-Rad Laboratories, LTD., Hemel Hempstead, UK) and normalized to the intensity of the housekeeping gene GAPDH. The background in the films was subtracted from the optical density measurements. The experiments were repeated three times, and the values that were obtained for the relative intensities were subjected to statistical analysis [29–33]. 

### 2.11. Statistical Analysis

All data were analyzed with a commercial statistical program (SPSS 16.0 for Windows, IBM Corporation, Armonk, NY, USA). Descriptive statistics were expressed as mean ± (standard deviation (SD)). The normal distribution of the data was assessed with a Shapiro-Wilk test. One-way analysis of variance (ANOVA) tests and Wilcoxon W tests were used to evaluate the differences in PT and the levels of expression of CB1 and dopamine receptors among the groups. Post-hoc comparisons, if applicable, were performed with Least Significant Difference tests. Statistical significance was accepted with *P* values less than 0.05 [31, 34]. Our analyses showed that the blinding procedure was applied successfully. Thus, both the evaluations of the results and the statistical analyses were conducted in a blind fashion.

## 3. Results

### 3.1. Cumulative Effects of Repeated EA on AA Rat's Behavioral Hypersensitivity

Thermal hyperalgesia of the hind paw stably occurred one day after the CFA injection [21]. EA that was delivered to the ipsilateral hind limb at the “ST36” and “BL60” acupoints was given 4 times during the experiment. The baseline measures of PWL to radiant heat stimulation on the left ankle articular cavity of the hind paw did not differ between the model and sham-model groups prior to the intra-articular injection.

**Figure 3:**
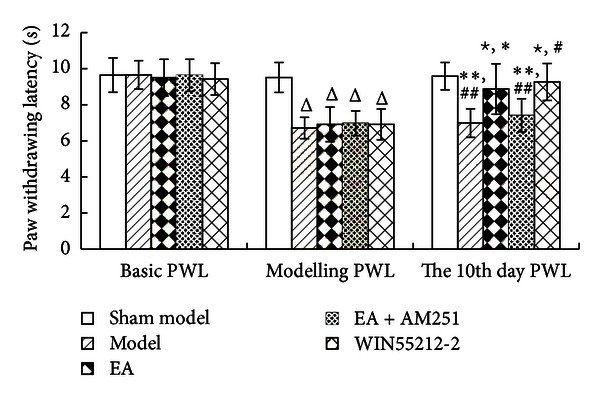
Paw withdrawing latency (PWL) values in the indicated groups. The data are presented as mean ± SD (*n* = 12). ^Δ^ *P* < 0.01 versus sham-model group at the same time-point. ^*☆*^ *P* < 0.01 versus model group at the same time-point. *P < 0.01 versus electroacupuncture (EA) group at modeling PWL. ^#^ *P* < 0.01 versus WIN55212-2 group at modeling PWL. **P < 0.01 versus EA group at the same time-point. ^##^ *P* < 0.01 versus WIN55212-2 group at the same time-point.

Following the unilateral intra-articular injections of CFA, strong thermal hyperalgesia developed within 1 day, and it persisted for over 10 days [17] in the ipsilateral hind paw (*P* = 0.00).The PWL was prolonged after repeated EA (*P* = 0.000), while the analgesic effects of EA were attenuated after an injection of AM251 (*P* = 0.001). There was no significant difference in the analgesic effects between the WIN55212-2 group and the repeated EA groups (*P* = 0.346) (Figure 3).

### 3.2. Upregulation of the Cannabinoid CB1 Receptor Protein Levels of Expression in the Striatum

**Figure 4:**
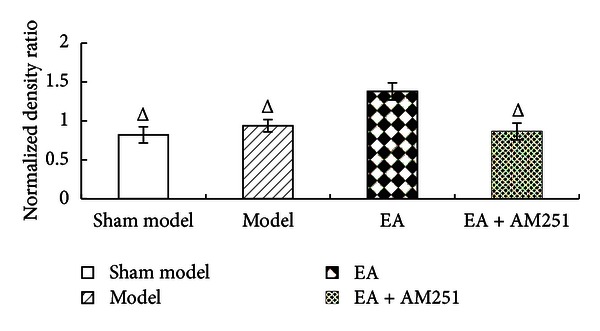
Normalized density ratio of CB1R and GAPDH in the nucleus accumbens and the caudate nucleus of each group (mean ± SD). ^Δ^ *P* < 0.01 versus EA group at the same time-point. CB1R, cannabinoid 1 receptor; GAPDH, glyceraldehyde 3-phosphate dehydrogenase.

**Figure 5:**
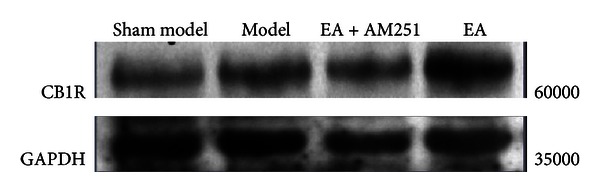
Comparison of the levels of expression of the CB1 receptor in the corpus striatum that were determined by western blot.

The results of the western blot analyses of the cannabinoid CB1 receptor protein levels demonstrated that there was no significant difference between the sham-model group and the model group (*P* = 0.190). The cannabinoid CB1 receptor protein levels were higher in the EA group than in the control group (*P* = 0.001). In addition, the CB1 receptor protein levels of the EA + AM251 group were notably lower than those in the repeated EA group (*P* = 0.000). There were no significant differences in the levels of protein expression of the CB1 receptor between the EA + AM251 group and the sham-model or the model groups (*P* = 0.391) (Figures 4 and 5).

### 3.3. Enhanced Effects of Repeated EA on the Upregulation of CB1 mRNA Expression in the Striatum of AA Rats

**Figure 6:**
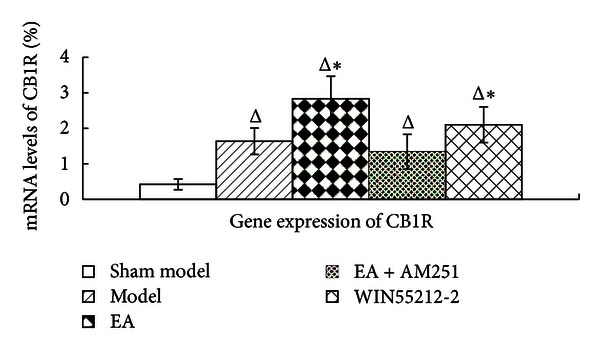
CB1 receptor gene expression in the corpus striatum in rats in the indicated groups. The data are presented as mean ± SD (*n* = 5). FQ-PCR, fluorescence quantitative-polymerase chain reaction. ^Δ^ *P* < 0.05 versus sham-model group at the same time-point. *P < 0.05 versus EA + AM251 group at the same time-point.

The results of the FQ-PCR analyses further demonstrated that the intra-articular injection of CFA produced a marked increase in the levels of CB1 receptor mRNA in the corpus striatum (*P* = 0.000; *P* = 0.000; *P* = 0.005; *P* = 0.000). Most interestingly, repeated EA resulted in a profound enhancement of the upregulation in the levels of CB1 receptor mRNA in AA rats (*P* = 0.001). AM251 markedly reduced the EA-induced upregulation in the CB1 receptor mRNA levels (*P* = 0.000). No significant difference was detected between the EA + AM251 and the model group (*P* = 0.319). The CB1-selective antagonist AM251 inhibited the analgesic effects of repeated EA by reversing the increase in the levels of expression of the CB1 receptor, suggesting that CB1 receptor probably acts as an important mediator of the anti-inflammatory role of EA (Figure 6).

### 3.4. Increased Effects of Repeated EA on the Upregulation of D1 mRNA Levels in the Striatum of AA Rats

**Figure 7:**
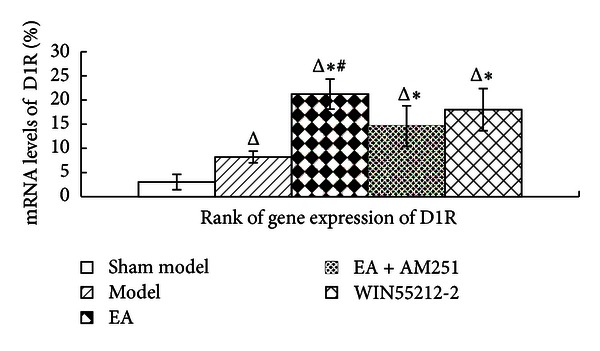
Rank of D1 receptor gene expression in the corpus striatum in the indicated groups. The data are presented as mean ± SD (*n* = 5). ^Δ^ *P* < 0.05 versus sham-model group at the same time-point. *P < 0.05 versus model group at the same time-point. ^#^ *P* < 0.05 versus EA + AM251 group at the same time-point.

In order to observe whether the cross-modulation of the dopamine system and cannabinoid CB1 receptor influenced the analgesic effects of repeated EA, dopamine D1 and D2-mRNA levels were determined with FQ-PCR analyses. The results revealed that an intra-articular injection of CFA resulted in a significant enhancement of dopamine D1 mRNA expression in the corpus striatum (*P* = 0.02; *P* = 0.000; *P* = 0.000; *P* = 0.000). Following EA treatment, the levels of expression of the D1 receptor mRNA were markedly increased (*P* = 0.000). An injection of AM251 resulted in a downregulation of the EA-induced effects (*P* = 0.005). Interestingly, there was no significant difference in the levels of D1 receptor mRNA expression between the repeated-EA group and the WIN55212-2 group (*P* = 0.135). Likewise, no significant difference was detected between the EA + AM251 and the WIN55212-2 groups (*P* = 0.113), indicating that AM251 had little impact on dopamine D1. In our experiment, different aspects of the changes in the expression of the dopamine D1 receptor and the cannabinoid CB1 receptor were observed. However, after an injection of WIN55212-2, D1 gene expression was significantly increased (*P* = 0.000), thus suggesting that, in the AA rat model, a strong activation of the CB1 receptor resulted in a significant change in the dopamine D1 receptor mRNA levels (Figure 7).

### 3.5. Increased Effects of Repeated EA on the Upregulation of D2 mRNA Levels in the Striatum of AA Rats

**Figure 8:**
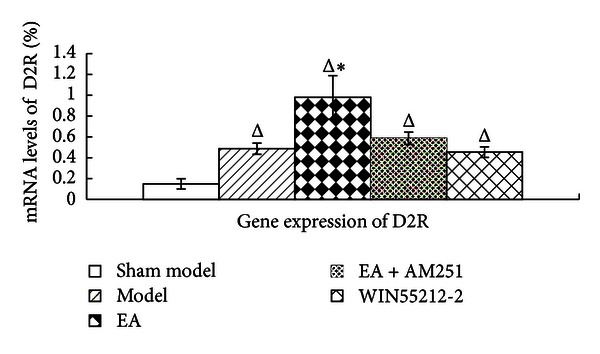
D2 receptor gene expression levels in the corpus striatum in the indicated groups. The data are presented as mean ± SD (*n* = 5). ^Δ^ *P* < 0.001 versus sham-model group at the same time-point. *P < 0.01 versus model group at the same time-point.

The changes in the dopamine D2 mRNA levels, which were similar to those of dopamine D1, were detected during FQ-PCR analyses. They showed that inflammatory pain resulted in a significant enhancement of the levels of dopamine D2 mRNA expression in the corpus striatum (*P* = 0.000; *P* = 0.000; *P* = 0.000; *P* = 0.000). Likewise, the levels of expression of D2 receptor mRNA were markedly increased (*P* = 0.000) following EA treatment. Interestingly, there was no significant difference in the levels of D2 receptor mRNA expression between the WIN55212-2 and the model groups (*P* = 0.624). AM251 resulted in a downregulation of the EA-induced effects (*P* = 0.000). According to the changes in dopamine D2 mRNA levels, the effects of repeated EA analgesia were different from the analgesic effects of WIN55212-2 (*P* = 0.000). Furthermore, regarding their mRNA levels, the dopamine D2 receptor displayed much more activation than the dopamine D1 receptor did (*P* = 0.000 versus *P* = 0.135, resp.) (Figure 8).

## 4. Discussion

To the best of our knowledge, this is the first time that a scientific investigation has demonstrated a relationship between repeated EA and cannabinoid CB1 receptor levels in the corpus striatum. In the present study, we established an AA rat model and evaluated the effects of repeated EA stimulation on the experimental arthritis. Our data showed that intra-articular injections of CFA resulted in a robust enhancement of the levels of gene expression of cannabinoid receptors and dopamine D1 and D2 receptors, suggesting that these three receptors are involved in the process of arthritis-induced hyperalgesia and allodynia. After repeated EA treatment, these target genes displayed much higher levels of expression. Furthermore, the combination of repeated EA with the CB1-selective antagonist AM251 resulted in a marked decrease in mRNA levels, indicating that the effects of repeated EA might be associated with an upregulation of cannabinoid CB1 receptors and that EA acts on the cross-talk of the cannabinoid CB1 receptors and the dopamine systems in the striatum. In addition, the analgesia resulting from repeated EA was different from the analgesic effects of WIN55212-2, which is a potent cannabinoid receptor agonist. Most interestingly, the EA-induced up-regulation of the levels of D2 gene expression was much greater than that of the levels of D1 gene expression. However, it was observed that the strong activation of the CB1 receptor after repeated EA might lead to the concomitant phenomenon of an up-regulation of the levels of D1 and D2 gene expression in the AA rat model. 

As a traditional Chinese medical technique, acupuncture has become very popular worldwide in the treatment of various illnesses. Our present data consistently indicated that repeated EA at acupoints, such as ST36 and BL60, could control inflammatory pain. Previous studies have also supported our results by showing that analgesic effects are exerted by EA stimulation at the ST36 and BL60 acupoints [9, 10, 35, 36].

Cannabinoid receptors are present not just in vertebrates but also in molluscs, leeches, and other invertebrate groups that have been evolutionarily separated for 500 million years [6]. The fact that natural selection has for so long conserved these receptors is an indication of their physiological importance. Cannabinoid actions in the central nervous system are confined to specific areas, most of which are involved in processing pain signals. One unproved but intriguing idea is that endocannabinoids may set the analgesic tone of the body, with the level of their production acting as a kind of pain thermostat. The use of acupuncture is controversial [37]. Numerous studies have indicated that EA stimulation is effective in relieving the pain of both somatic and visceral origin in humans and animals [38–40], and the ventrolateral column of the periaqueductal gray (PAG) may play a key role in the integration of the physiological responses to somatic and visceral pain [41]. Similar to previous studies, the posterior ventrolateral PAG is considered an important brain area for the antinociceptive effects of cannabinoids [6, 42, 43]. 

The functional interactions between endocannabinoid and dopaminergic systems may contribute to striatal signaling [44, 45]. CB1 receptors are colocalized with D1 or D2 receptors on the same population of striatal membranes, and they can interact at the level of G-protein/adenylyl cyclase signal transduction [46]. The endogenous cannabinoid system is involved in regulating striatal dopamine release [47]. The activation of postsynaptic dopamine receptors controls endocannabinoid mobilization by acting on presynaptic CB1 receptors and thus modulating glutamate release differently in glutamate terminals that project to D1 and D2 cells [48]. However, the endocannabinoid system is unlikely to directly affect dopamine release. Instead, it can modify dopamine transmission through transsynaptic mechanisms. These mechanisms involve gamma-aminobutyric acid (GABA) ergic and glutamatergic synapses, as well as converging signal transduction cascades of the cannabinoid and dopamine receptors. The dopamine and endocannabinoid systems exert mutual control on each other [49].

In the striatum, CB1 receptor activation has been shown to promote long-lasting changes in synaptic activity at both corticostriatal and striatonigral/pallidal synapses [50–52]. This activation also increases both the levels of extracellular dopamine and the activity of dopamine neurons [53, 54]. Cannabinergic signalling may lead to the release of dopamine, which can act through dopamine D1-like receptors as a negative feedback mechanism to counteract the effects of the activation of the cannabinoid CB1 receptor [49]. The elimination of dopamine D1 receptors has opposite effects on cannabinoid-mediated ERK1/2 signaling [55]. In addition, dopaminergic signaling through dopamine D2-like receptors may lead to the up-regulation of cannabinergic signaling, which is likely to represent a negative feedback on dopaminergic signaling [49]. 

**Figure 9:**
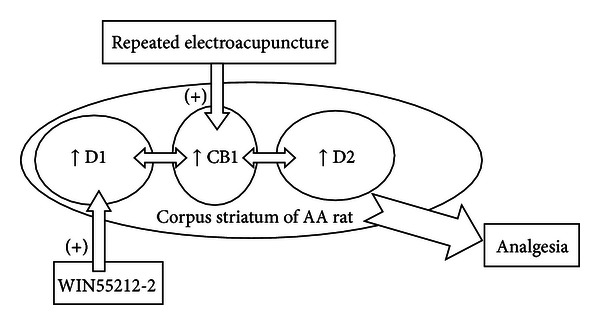
A hypothetical diagram illustrating the EA action that is mediated by CB1 in the adjuvant arthritis (AA) rat. The intra-articular injection of Complete Freund's adjuvant (CFA) promotes the expression of CB1, D1, and D2. EA stimulation at specific acupoints results in a much higher expression of these three receptors, but the CB1 receptor antagonist AM251 can reverse these antinociceptive effects. Compared to the action of WIN55212-2, EA mainly produces its effects by upregulating the cross-modulation of dopamine receptors and CB1 in the corpus striatum, while WIN55212-2, which is a potent CB1 receptor agonist, exerts its function on the relationship between CB1 and D1 for pain relief.

CB1 receptors that are located in the corpus striatum are involved in a number of biological processes, including analgesia [50], addiction [56], and motor deficits [46]. The corpus striatum has always been regarded as an active area that is related to EA analgesia [14]. Our results demonstrated that the CB1-selective antagonist AM251 could attenuate the analgesic effects of repeated EA by reversing the increased expression of the CB1 receptor, suggesting that the CB1 receptor plays an important role in EA analgesia. The two kinds of dopamine receptors have different influences on the EA-induced CB1 up-regulation in the AA rat model. The change in the EA-induced up-regulation of the levels of D2 gene expression was much greater than that of the D1 gene expression. It was also observed that the strong activation of the CB1 receptor after repeated EA might lead to the concomitant phenomenon of the up-regulation of the D1 and D2 levels of gene expression in the AA rat model. A hypothetical diagram of the anti-inflammatory actions of EA that are mediated by the cross-modulation of CB1 and the dopamine receptors in the AA rat model is shown in Figure 9.

In conclusion, our results indicated, for the first time, that EA treatment at Zusanli (ST36) and Kunlun (BL60) produced strong antinociceptive effects in rats with CFA-induced arthritis, at least partially, through an up-regulation in the levels of the cannabinoid CB1 receptor expression. The relationship between CB1 receptors and dopamine receptors may play a role in the analgesia that is associated with repeated EA. In addition, the analgesic effects of repeated EA were different from the analgesic effects of the CB1-selective agonist WIN55212-2. While WIN55212-2 exerted its function on the relationship between CB1 and D1 for pain relief, EA wields its effect by upregulating the cross-modulation of dopamine receptors and CB1 in the striatum. Thus, repeated EA analgesia had a more complex mechanism.

## Figures and Tables

**Table 1:** Sequences of primers specific for real-time polymerase chain reaction analyses.

Target sequence	Sequence (5′ → 3′)	Size (bp)
Glyceraldehyde 3-phosphate dehydrogenase (GAPDH)	R: CGAGGGCCCACTAAAGG	116
	F: GCTGTTGAGTCACAGGAGCAA	
Cannabinoid receptor 1 (CB1)	R: TATCGCAATAGTAATCGCTGTGTTG	182
	F: GTACATTCTCTGGAAGGCTCACA	
Dopamine 1 receptor (D1)	R: GGGACTCCAACTCACTCTGC	187
	F: CTACATGGAGCCCGAGAAGC	
Dopamine 2 receptor (D2)	R: GCCAACCTGAAGACACCACTC	159
	F: GGAAATGGAGATGCTGTCAAGC	
